# A name unasked

**DOI:** 10.1017/S1478951526101710

**Published:** 2026-01-28

**Authors:** Shehraz Riar

**Affiliations:** Faculty of Health Sciences, McMaster University, Hamilton, Ontario, Canada

My name is always

read from the band.

Still.

They smooth it out.

A corner rounded,

not for function,

but speed.

I have done that, too.

All my life.

Through doors,

at counters,

under bright lights.

I learned to be cut into a million pieces.

So that others could stay whole.

So that my children could go through

without apology.

Now the bed rails hold me.

Unlike any other border.

Unlike any other regime.

Voices move around:

clean, practiced, precise.

Full of words that land

like a drum in my chest,

but thin out

near the crown.

Another one in scrubs asks,

“Any pain?”

Yet pain is not my first language.

In my first language

pain is not a number.

It is a season.

It has a smell.

It brings loved ones along with it.

My mouth tries the right syllables,

but to the left it fails.

To the surprise of none.

I think of lunches packed

before dawn.

The lid snapped shut

so tight

nothing could spill.

A whole life

of feeding others first –

and now

I am fed by strangers

who do not know

what comfort tastes like.

My tongue remembers

cardamom,

hot tea,

the soft burn of ginger –

the way my mother

could make a room feel safe

with steam.

Here,

everything is measured

except that.

The nurse comes closer.

She does not rush

to fix me.

In that quiet,

my breathing changes.

Not cured.

Not saved.

Just,

less alone.

And for the first time in years

I do not cut corners.

I let my name

remain whole.

